# Response of Primary Human Adipocytes to Fatty Acid Treatment

**DOI:** 10.1111/jcmm.70622

**Published:** 2025-05-26

**Authors:** Katarzyna Pietraszek‐Gremplewicz, Joanna Olszańska, Mikołaj Domagalski, Aleksandra Simiczyjew, Magdalena Kot, Aneta Skoniecka, Agata Tymińska, Michał Pikuła, Dorota Nowak

**Affiliations:** ^1^ Department of Cell Pathology, Faculty of Biotechnology University of Wroclaw Wroclaw Poland; ^2^ Laboratory of Tissue Engineering and Regenerative Medicine, Division of Embryology Medical University of Gdansk Gdansk Poland

**Keywords:** adipocytes, fatty acids, lipid droplets, obesity

## Abstract

Obesity, nowadays a common disease, due to its complexity can cause many other disorders. In this study, a model of preadipocytes isolated from human adipose tissue was used. Cells after differentiation were additionally fattened with fatty acids such as palmitic, oleic and linoleic. Compared to control cells, obtained cells constitute a strongly reliable research model that mimics obesity occurring in humans. Achieved results have shown that adipocytes treated with fatty acids exhibited a greater number of both ‘large’ and ‘small’ lipid droplets, and an increase in lipid droplet formation and maintenance‐related proteins, elevated expression of genes encoding proteins involved in the transport of fatty acids and raised secretion of cholesterol and glutamate. Fattening has also resulted in changes in the phenotype of vimentin filaments and actin cytoskeleton reorganisation. Finally, it has also been observed that hypertrophy of adipocytes was accompanied by modifications in cell metabolism, phenotypic and quantitative changes in mitochondria with a simultaneous downregulation of genes involved in mitochondrial fusion. In summary, the human research model that we propose allowed us to demonstrate several adjustments in cells mimicking the obesity state, which may contribute to expanding knowledge about obesity and improving treatment strategies for this disease.

## 1 Introduction

The obesity condition is one of the most noticeable and under‐appreciated public health problems that increase the risk of multiple pathological states, such as cardiovascular diseases, musculoskeletal disorders, metabolic syndrome, insulin resistance, diabetes and selected types of cancer. The causes of those diseases are complex and multi‐factorial. Obesity may be related to an energy imbalance between calories consumed and expended, resulting in enhanced fat storage [1]. This caloric inequality is strictly related to the increasing dysfunction of adipose tissue (AT) physiology. AT is composed of heterogeneous cellular populations, with adipocytes as the main component [2]. Adipocytes, aside from being lipid‐rich cells that mainly store long‐chain fatty acids (FAs) in the form of lipid droplets (LDs), are also an active source of a wide array of effectors including exosomes, miRNA, lipids and bioactive molecules called adipokines. Therefore, they may act not only as local paracrine signalling cells but also at distant levels through the secretion of molecules into the circulation and induction of systemic metabolic responses [3].

Even though overweight and obesity are multi‐factorial conditions, the biological role of adipocytes may be addressed as a tool to understand the cause and the search for therapeutic approaches. At the cellular level, prolonged caloric excess shifts metabolic activity of adipocytes from energy supply to lipid storage [4]. Under these conditions, adipose depots can enlarge, with new adipocytes differentiating from preadipocytes in the process of adipogenesis (hyperplasia). However, the main contributor to the growth of AT is hypertrophy, that is, the increase in the volume of adipocytes [5]. This biological process is correlated with metabolic complications involving dysfunction of membrane proteins, loss of mitochondrial biogenetic capacity, dysregulation of lipid, amino acid, glucose and nucleotide metabolism, alterations in autophagy and adipokine secretion [4, 5].

Similar to other areas of biomedical research, rodents (rats and especially mice) are widely used as preclinical animal models to study the underlying mechanisms of obesity [6]. Similarly, the in vitro research is most often based on the mouse 3T3‐L1 cell model, which under appropriate conditions, acquires an adipocyte‐like phenotype [7]. Then, they could be additionally fattened with unsaturated (oleate and linoleate), as well as saturated (myristic, palmitic and stearic) FAs. These FA‐overloaded cells were used in several studies as a model to mimic features of obese adipocytes [8, 9]. However, it is obvious that in vitro and in vivo animal models cannot fully imitate human cells because of the physiological and metabolic differences between species. From all these reasons, a model simulating obesity in humans is being searched. Preadipocytes and adipose‐derived stem cells isolated from AT are the most common examples enumerated in the literature for studying hyperplasia of AT [7]. Nevertheless, there are relatively few studies examining the hypertrophy of human cells, such as mesenchymal stem cells [10], preadipocytes [11] or adipose‐derived stem cells [12]. A common feature of this research is the supplementation of adipocytes with only one type of FA. To address this need in our studies, we used human adipocytes fattened by incubation with three different types of FAs to mimic obesity conditions. This allows us to generate a model of human hypertrophic adipocytes directly comparable to normal adipose cells. Next, we evaluated the phenotypic and functional differences between control and those incubated with FAs adipocytes.

## 2 Material and Methods

### 2.1 Material

Human subcutaneous AT was obtained from patients at a plastic surgery clinic (Gdansk, Poland). All procedures were conducted following informed consent from patients and with the approval of the Bioethics Committee for Scientific Research of the Medical University of Gdansk (NKBBN/858/2022‐2023). The tissue samples were considered medical waste following aesthetic body contouring procedures. Adipose tissue samples were collected from eight healthy female donors with an age range of 30–44 years.

### 2.2 Isolation and Culture of Adipose Tissue‐Derived Mesenchymal Stromal Cells (AD‐MSCs)

The isolation was based on previously described protocols [13]. Initially, the tissue was minced into small fragments and treated with collagenase I solution (Sigma‐Aldrich) in a 37°C water bath to facilitate the digestion of extracellular matrix components. Subsequent steps involved the separation of cells using a mesh filter, followed by erythrocyte lysis, washing and centrifugation. The freshly isolated cells were seeded in Dulbecco's Modified Eagle medium (DMEM, IITD PAN, Wrocław, Poland) containing 1 g/L glucose supplemented with 10% foetal bovine serum (FBS) (Sigma‐Aldrich) and antibiotics–antimycotic solution (100 U/mL penicillin, 100 μg/mL streptomycin and 0.25 μg/mL amphotericin B) (Gibco). Daily medium changes were performed to remove non‐adherent cells, enriching the culture with AD‐MSCs. Cells were cultured at 37°C in 5% CO_2_ in a humidified atmosphere.

### 2.3 Adipocyte Differentiation and Incubation With FAs

Human adipocytes were obtained from AD‐MSCs according to the protocol previously described by Wang et al. [14]. Cells at passage 3 were used for experiments. Briefly, 24 h after reaching 100% confluence, cells were incubated with differentiation medium (DM), which was composed of DMEM/F12 medium (Gibco) supplemented with 10% FBS (Gibco), 2 mM glutamine (Sigma‐Aldrich), antibiotics–antimycotic solution (100 U/mL penicillin, 100 μg/mL streptomycin and 0.25 μg/mL amphotericin B) (Gibco), 5 μg/mL insulin, 0.5 mM 3‐isobutyl‐1‐methylxanthine (IBMX), 5 μM dexamethasone and 200 μM indomethacin (all from Sigma‐Aldrich). Differentiation of AD‐MSCs took 14 days. After 7 days, the DM was replaced with the fresh one for the next 7 days.

After differentiation, adipocytes were incubated with FAs as previously described by Balaban et al. [15]. Briefly, after 14 days, DM was changed to DMEM/F12 medium (Gibco) supplemented with 10% FBS (Gibco), 2 mM glutamine (Sigma‐Aldrich), antibiotics–antimycotic solution (100 U/mL penicillin, 100 μg/mL streptomycin and 0.25 μg/mL amphotericin B) (Gibco), 0.33 mM linoleate, 0.17 mM palmitate and 0.17 mM oleate (all from Sigma‐Aldrich) for 48 h. Control cells were incubated in a DMEM/F12 medium (Gibco) supplemented with 10% FBS, 2 mM glutamine, antibiotics–antimycotic solution (100 U/mL penicillin, 100 μg/mL streptomycin and 0.25 μg/mL amphotericin B) and containing 0.44% bovine serum albumin (BSA, Sigma‐Aldrich) and 0.26% ethanol, which were also used as FAs solvents. All experiments were performed with cells isolated from at least three different donors.

### 2.4 Immunocytochemistry

The subcellular distribution of cell nuclei, LDs, F‐actin, G‐actin and vimentin was examined using fluorescence confocal microscopy. Cells cultured on coverslips were fixed with 4% formaldehyde and permeabilised with 0.1% Triton X‐100 in PBS. Non‐specific binding was blocked by 1% BSA in PBS. Lipid droplets were visualised using LipidSpot 488 (Biotium), mitochondria with MitoTracker Orange CMTMRos (Invitrogen), nuclei with Hoechst 33342 reagent (Invitrogen), actin filaments with Phalloidin CruzFluor 488 Conjugate (Santa Cruz Biotechnology) and G‐actin with Alexa Fluor 594‐labelled DNase I (ThermoFisher Scientific). Cytoplasm was labelled with high‐content screening CellMask Deep Red Stain (Invitrogen). Rabbit anti‐vimentin antibody (GeneTex) followed by Alexa Fluor 488‐conjugated anti‐rabbit secondary antibody (Invitrogen) was applied to visualise this protein. Confocal images for quantitative measurements were acquired using an Opera Phenix Plus System (Perkin Elmer) and calculated by dedicated Harmony software. The data were determined for at least 500 cells in each condition and repetition. The vimentin expression was quantified as the sum of the intensity of the positive pixels and normalised to the number of nuclei present in the image area. Similarly, the F:G actin ratio was calculated based on the quantification of F and G actin fluorescence intensity and next divided by the number of nuclei. For the more precise subcellular structures analysis, the confocal images were captured using a Leica Stellaris 8 (Leica, Wetzlar, Germany) and LAS X software (ver. 3.3.0, Leica, Wetzlar, Germany). For each condition, representative areas are shown.

### 2.5 qRT‐PCR Analysis

To evaluate the expression level of selected genes in adipocytes after incubation with FAs, RNA was isolated using Fenozol mixture (A&A Biotechnology) according to the manufacturer's protocol. Next, after on‐column digestion with DNase I (EURx), RNA was used for the reverse transcription reaction performed with a High‐Capacity cDNA Reverse Transcription Kit (Applied Biosystems) according to the producer's instructions. Quantitative PCR was done with PowerUp SYBR Green Master Mix. All results were normalised to the *hypoxanthine phosphoribosyltransferase 1* (*HPRT1*) gene based on ΔΔCT method. All primers, whose sequences were listed in Table 1, were purchased from Merck.

**TABLE 1:** Sequence of utilised primers.

Abbreviation	Full name of the gene	Forward primer 5′–3′	Reverse primer 5′–3′
*HPRT1*	*Hypoxanthine phosphoribosyltransferase 1*	gaccagtcaacaggggacat	gcttgcgaccttgaccatct
*PLIN1*	*Perilipin* *1*	gagctgaaggacaccatctc	gtactccaccaccttctcaatg
*PLIN2*	*Perilipin 2*	ccctacctgaagtctgtgtgtgag	aggcagtctctcctcaatcctg
*FABP4*	*Fatty acid binding protein 4*	gattatatgaaagaagtaggagtgggctt	ccatctaaggttatggtgctcttgac
*FABP5*	*Fatty acid binding protein 5*	gcagctggaaggaagatggc	aacttctctcccagggtacaagaa
*CD36*	*Cluster of differentiation 36*	ctaatgccagttggagacctgc	gctgctgttcatcatcacttcct
*CHREBP*	*Carbohydrate‐responsive element‐binding protein*	tccgacatctcagacactc	agatgtccatgaagtcatcc
*LPCAT2*	*Lysophosphatidylcholine acyltransferase‐2*	gatggcagcattgacttccgag	cctccgttatgtagccatcctc
*SCD 1*	*Stearoyl‐CoA desaturase 1*	tataccaccaccaccaccattacag	catattcaaccttggggcttggg
*FADS 1*	*Fatty acid desaturase 1*	tacctgctgcacatcttgct	atgtagcagatggttccactttgag
*GLUT1*	*Glucose transporter 1*	cagcaagaagctgacgggt	cagaaaagatggccactgagagg
*GLUT3*	*Glucose transporter 3*	atggggacacagaaggtcac	cggaaaatatggccacagaca
*GLUT4*	*Glucose transporter 4*	ctgatgactgtggctctgct	gaagagctcggccacgatg
—	*Adiponectin*	ttgctgggagctgttctact	tggatctcctttctcacccttc
—	*Caveolin‐1*	gaagcaagtgtacgacgc	caaagagggcagacagcaag
*FATP 1*	*Long‐chain fatty acid transport protein 1*	tgctgcagctccatgtgac	ctgacagtggtgacatccaagt
*OPA1*	*Optic atrophy 1*	ctgtgaggtctgccagtcttta	ctgtccttaattggggtcgttg
*MIEF1*	*Mitochondrial elongation factor 1*	caggatgacaatggcattggc	ccgatcgtacatccgcttaact
*MFN2*	*Mitofusin 2*	acatggagcgttgtaccagc	ttgagcacctccttagcagac
*MFF*	*Mitochondrial fission factor*	caaggttccaggcaccgatt	cgacaaaatgccacgagcag

### 2.6 Western Blotting Analysis

To collect protein lysates, cells were transferred on ice, washed three times with PBS and lysed with urea buffer (50 mM Tris, pH 7.4, 5% SDS, 8.6% sucrose, 74 mM urea and 1 mM dichlorodiphenyltrichloroethane) supplemented with protease and phosphatase inhibitor cocktails (Sigma‐Aldrich). The protein concentration in samples was measured by a standard bicinchoninic acid procedure (Thermo Fisher). The samples with the same amounts of protein were separated by polyacrylamide gel electrophoresis in the presence of sodium dodecyl sulfate (SDS‐PAGE) as reported by Laemmli [16] and then transferred to nitrocellulose membranes as reported by Towbin et al. [17]. Primary antibodies directed against CD36 (Abcam; ab133625), perilipin 1 (Cell Signaling; 9349T), perilipin 2 (Cell Signaling; 45535), Src (Millipore; 05‐184) and pSrc (SantaCruz; sc‐166860) as well as goat anti‐rabbit and anti‐mouse secondary antibodies conjugated with horseradish peroxidase (Cell Signaling) were used. Signals were detected with Clarity Western ECL Substrate (Bio‐Rad) using ChemiDoc (Bio‐Rad) and analysed with ImageLab Software (ver. 6.0, Bio‐Rad). All results were normalised to the total protein amount estimated by Ponceau S staining. Representative blotting membranes are shown.

### 2.7 Evaluation of Cholesterol and Glutamate Secretion Level

The level of cholesterol and glutamate secretion by adipocytes to the culture medium was assessed by the Cholesterol/Cholesterol Ester‐Glo and Glutamate‐Glo Assays (both from Promega), respectively. The experiment was executed according to the manufacturer's procedure, and luminescence was detected using the GloMax Discover plate reader (Promega). The luminescence value was calculated relative to the control conditions.

### 2.8 Adipokine Array

The adipokines secreted by adipocytes were analysed using the Human Adipokine Antibody Array (R&D Systems). The tests allow for the detection of 58 obesity‐related molecules in a single sample, thanks to spots of antibodies present on the nitrocellulose membranes. The experiment was conducted with conditioned media collected from control adipocytes and adipocytes incubated with FAs according to the manufacturer's protocol. The chemiluminescence signal was measured using a ChemiDoc Imaging System (Bio‐Rad) and analysed with ImageLab software (ver. 6.0, Bio‐Rad). Densitometric values were background corrected and then normalised to the mean of reference spots for each membrane.

### 2.9 ATP Production Rate

The cell metabolic status was investigated on a Seahorse XF Pro extracellular flux analyzer (Agilent Technologies) with standard 96‐well Seahorse microplates. A Seahorse XF Real‐Time ATP Rate Assay kit (Agilent Technologies) was applied following the manufacturer's instructions. The oxygen consumption rate and the extracellular acidification rate were measured. The assay was done in non‐buffered DMEM containing 10 mM glucose, 2 mM glutamine and 1 mM pyruvate. The injection sequence was programmed as follows: first, oligomycin (1 μM at final concentration); second, rotenone and antimycin A (0.5 and 0.5 μM at final concentrations, respectively). At the end of the Seahorse measurements, cell nuclei were stained with Hoechst 33342 (Invitrogen), next cells were imaged and counted by Citation 5 (Agilent BioTek). Obtained results were directly used to normalise the Seahorse parameters per cell number. The data were calculated by the Seahorse Analytics (Version: 1.0.0‐720, Agilent Technologies) as the ATP production rate in pmol/min/1000 cells and presented as the per cent of the control.

### 2.10 Statistical Analysis

All data are shown as the mean ± standard deviation (SD). Significance was established with GraphPad Prism 7 software. An unpaired *t*‐test with Welch's correction was applied. Significance was set at *p* < 0.05 (*), *p* < 0.01 (**), *p* < 0.001 (***) or *p* ≤ 0.0001 (****).

## 3 Results

**FIGURE 1:**
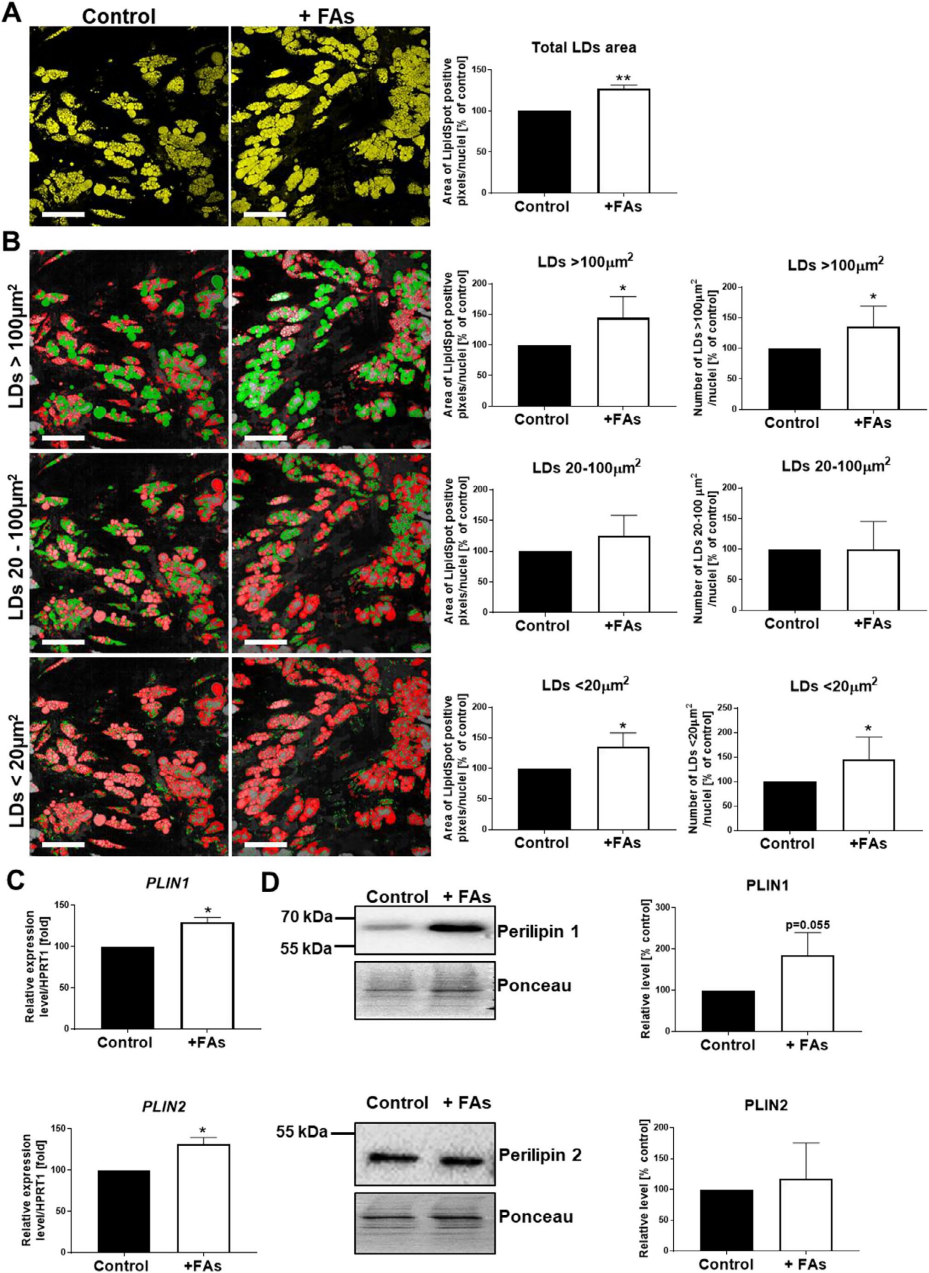
Effect of FAs on lipid accumulation in adipocytes. (A) Representative images of control and incubated with FAs adipocytes stained for neutral lipids (Lipid Spot 488 marked in yellow) with quantification of all positive signals corresponding to total LDs area. (B) The example images of the evaluation of area and number of LDs divided into three groups: > 100, 20–100 and < 20 μm^2^. The analysed LDs in the given fraction were marked in green, whereas the rest were marked in red. Scale bar: 200 μm. (C) mRNA and (D) protein level of PLIN1 (perilipin1) and PLIN2 (perilipin2) in control and treated with FAs adipocytes established by qRT‐PCR and Western blotting analysis, respectively. mRNA expression levels of target genes of interest were normalised to the mRNA expression of *HPRT1* (qRT‐PCR) and to total protein content assessed by Ponceau S staining (Western blotting). Representative blotting membranes are presented. In the case of quantitative analyses, the mean of at least three biological repetitions ± SD is shown. Asterisks indicate statistically significant differences at the level *p* ≤ 0.05 (*) and *p* ≤ 0.01 (**).

To elaborate on the influence of FAs on AT cells, we used human adipocytes obtained by differentiation of AD‐MSCs isolated from subcutaneous depots of healthy donors. Since obesity primarily leads to hypertrophy of adipocytes due to massive accumulation of LDs [8, 9], at first, we decided to evaluate the lipid content in cells incubated with FAs. Our results indicate that fattened adipocytes accumulated an increased amount of neutral lipids in comparison to control ones (Figure 1A). Next, based on microscopic images, we estimated the size of accumulated LDs. Adipocytes incubated with FAs had not only a higher number of ‘large’ (> 100 μm^2^) but also ‘small’ (< 20 μm^2^) LDs, and these compartments in both of these fractions covered bigger areas in the cytoplasm in comparison to control cells (Figure 1B). Moreover, fattened cells exhibited higher, than in control, mRNA expression of genes encoding *perilipin 1* and *2* (Figure 1C), as well as protein level of perilipin 1 (Figure 1D). These molecules are involved in LD formation and maintenance [18].

**FIGURE 2:**
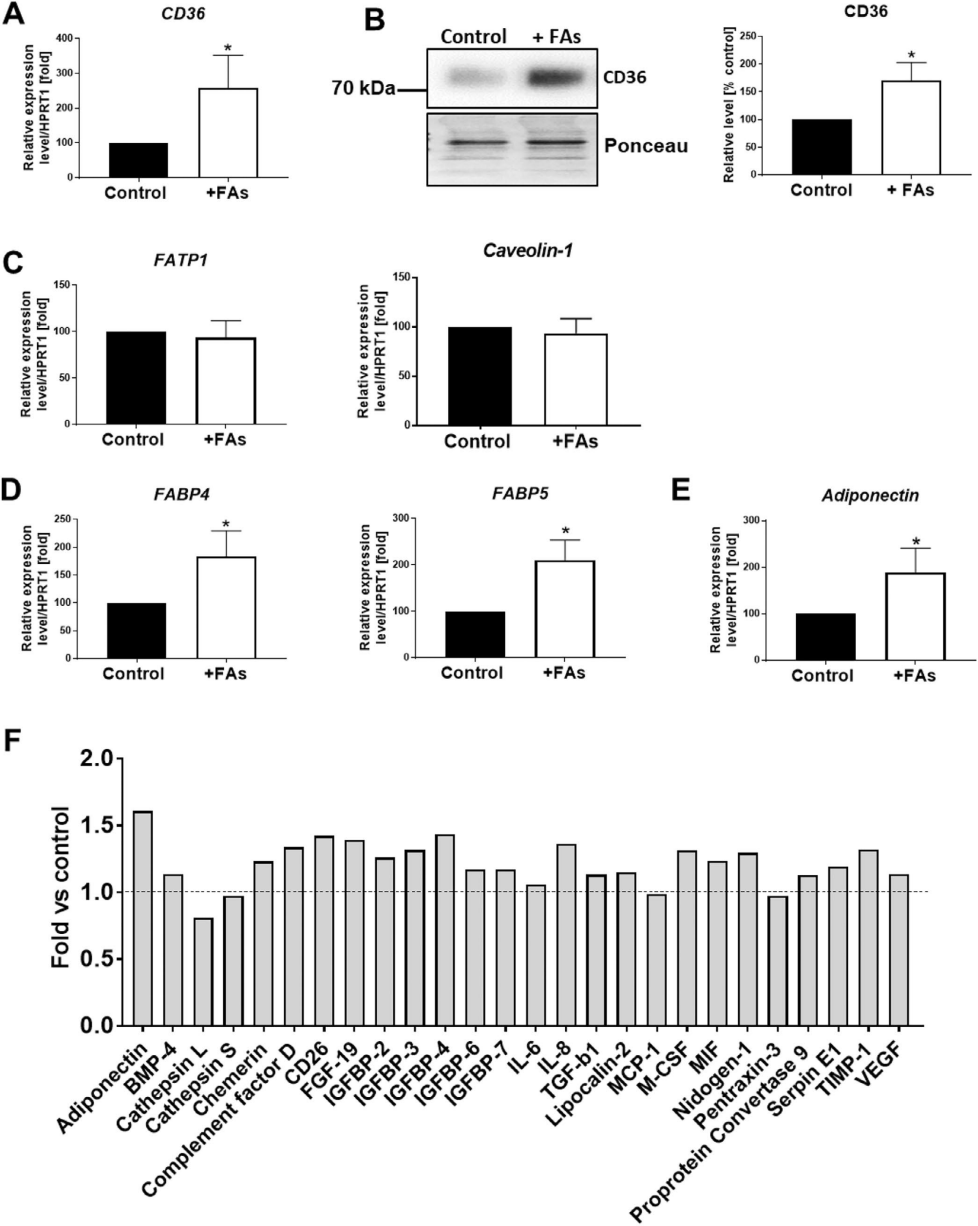
The influence of FAs on the expression level of fatty acid transporters and secretion of adipokines. (A) qRT‐PCR and (B) Western blotting analysis of CD36 level normalised to the expression of *HPRT1* and total protein content (Ponceau S staining), respectively. Representative blotting membranes are shown. qRT‐PCR assessment of (C) *FATP1*, *caveolin‐1*, (D) *FABP4, FABP5* and (E) *adiponectin* level normalised against the expression of *HPRT1*. The results from at least three biological repetitions were normalised to control and are presented as the mean ± SD. The significance level was set at *p* ≤ 0.05 (*). (F) Adipokines secreted to culture media detected by antibody array (*n* = 1). Based on obtained signals, quantitative analysis was conducted. Results were normalised to reference spots and are shown as a fold change versus control conditions. BMP‐4, bone morphogenetic protein 4; FGF‐19, fibroblast growth factor 19; IGFBP‐2, insulin‐like growth factor‐binding protein 2; IGFBP‐3, insulin‐like growth factor‐binding protein 3; IGFBP‐4, insulin‐like growth factor‐binding protein 4; IGFBP‐6, insulin‐like growth factor‐binding protein 6; IGFBP‐7, insulin‐like growth factor‐binding protein 7; IL‐6, interleukin 6; IL‐8, interleukin 8; MCP‐1, monocyte chemoattractant protein 1; M‐CSF, macrophage colony‐stimulating factor; MIF, macrophage migration inhibitory factor; TGF‐β1, transforming growth factor beta 1; TIMP‐1, tissue inhibitor of metalloproteinases 1; VEGF, vascular endothelial growth factor.

Long‐chain FAs can enter cells by a few mechanisms, like passive diffusion and translocation across the plasma membrane by transporters (CD36, FA transport protein [FATP] and caveolin‐1). Next, these molecules that are taken up by cells bind to FA‐binding proteins (FABPs) and are transported to the specific organelles [19]. A higher expression of CD36 mRNA (Figure 2A) and protein (Figure 2B), as well as upregulation of mRNA encoding *FABP4* and *5* (Figure 2D), was observed in fattened cells compared to control ones. No differences were noted in the mRNA expression level of *FATP1* and *caveolin*‐*1* (Figure 2C). Since the marker of mature adipocytes, adiponectin, was demonstrated to be regulated by CD36 [20], in the next step we evaluated the expression level of this adipokine. Adipocytes treated with FAs expressed a higher level of mRNA of *adiponectin* than control cells (Figure 2E). Because adipocytes are not only fat reservoirs, but also a prominent source of biologically active molecules, the achieved result prompted us to broaden our analysis. An antibody‐based array revealed moderate modifications in the profile of adipokines secreted by fattened adipocytes in comparison to the control ones (Figure 2F). Secretion of most of the detected factors was moderately upregulated, including several molecules from the insulin‐like growth factor‐binding protein (IGFBP) family and cytokines (such as interleukin 8 [IL‐8], macrophage colony‐stimulating factor [M‐CSF] and macrophage migration inhibitory factor [MIF]). Interestingly, the highest increase was detected for adiponectin confirming on protein level the result from qRT‐PCR evaluation.

**FIGURE 3:**
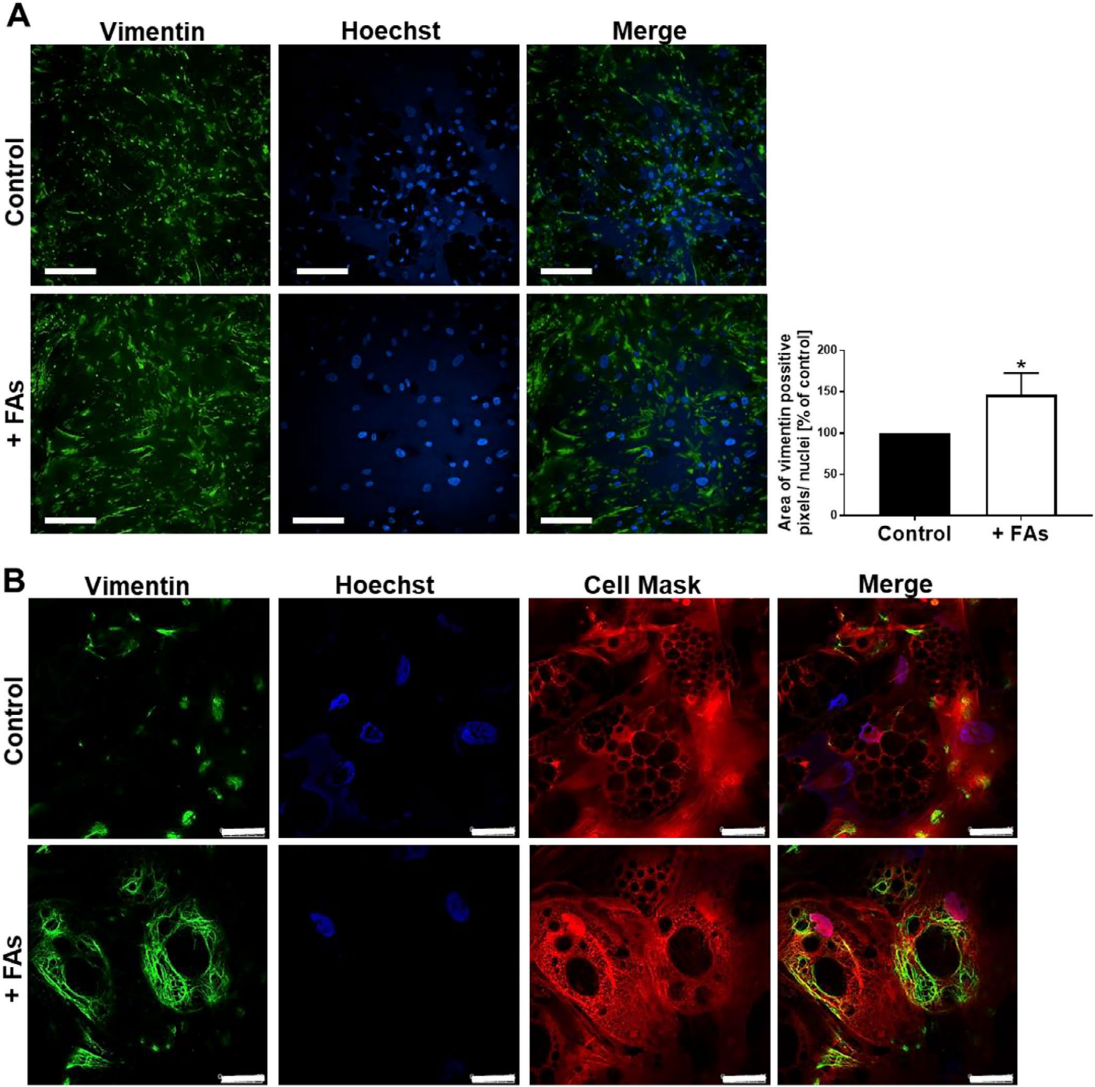
Higher vimentin protein levels in adipocytes incubated with FAs. (A) Representative images of cells stained for vimentin (green) and cell nuclei (blue) taken using high‐content screening confocal microscope with quantification of pixels corresponding to vimentin expression. Results are presented as the mean from at least three independent experiments ± SD. *p* ≤ 0.05 (*). Scale bar: 200 μm. (B) Representative image of cellular distribution of vimentin (green), cell nuclei (blue) and cytoplasm (Cell Mask, red). Scale bar: 25 μm.

Previous studies correlated CD36 expression with the level of vimentin [21], which is the only intermediate filament protein expressed in preadipocytes and may participate in adipogenesis through LD formation or homeostasis [22]. Since adipocytes incubated with FAs demonstrated a higher content of LDs, we explored vimentin distribution in analysed cells. Whole‐population images showed that fattened adipocytes possess a higher level of vimentin (Figure 3A). Moreover, a network formed by vimentin fibres was more developed, which was confirmed by the more precise microscopic observations using greater magnification. Fatty acids induced the formation of a system composed of long vimentin filaments, which covered the entire cytoplasm of cells (Figure 3B).

**FIGURE 4:**
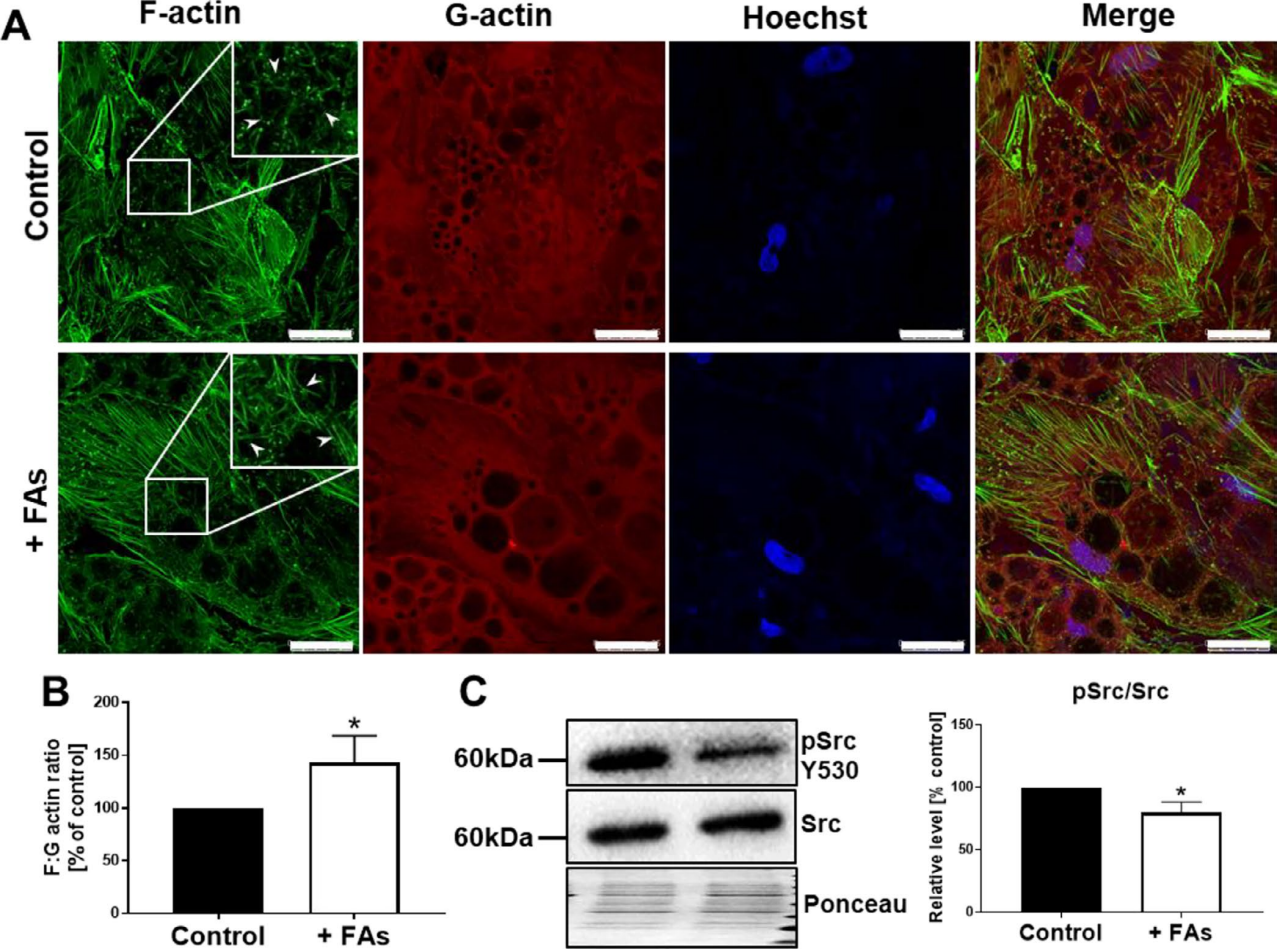
Remodelling of actin cytoskeleton in fattened adipocytes. (A) Representative images of cells stained for F‐actin (green), G‐actin (red) and cell nuclei (blue) with (B) a calculation from the high screening microscopy of F:G actin ratio based on quantification of fluorescence intensity. Insets show an enlarged view of the boxed areas. Arrowheads indicate the near surroundings of LDs. Scale bar: 25 μm. (C) Western blotting analysis of autoinhibitory pSRC (Y530)/SRC ratio normalised to total protein content (Ponceau S staining). Representative blotting membranes are shown. All results are presented as the mean ± SD from at least three independent experiments and were calculated as the percentage of control *p* ≤ 0.05 (*).

Another element of the cell cytoskeleton whose dynamics can be modified in fattened adipocytes is the actin network. It was shown that the expansion of adipocytes from high‐fat diet‐fed mice was associated with a drastic increase in filamentous (F)‐actin [23]. Thus, we analysed F‐ and globular (G)‐actin levels in our experimental model. Hypertrophic adipocytes accumulated short, thin actin fibres in a close proximity to LDs, which was not detected in the case of control cells, containing smaller LDs (Figure 4A, insets). This observation after quantification was reflected in an increased F:G ratio in these cells (Figure 4B). One of the factors controlling actin dynamics is Src kinase, whose activity enhances actin assembly [24]. Results of Western blotting analysis demonstrated that the treatment of adipocytes with FAs decreased the phosphorylation of the C‐terminal tail of Src (Figure 4C), which bears an autoinhibitory phosphorylation site at tyrosine 530 [25].

**FIGURE 5:**
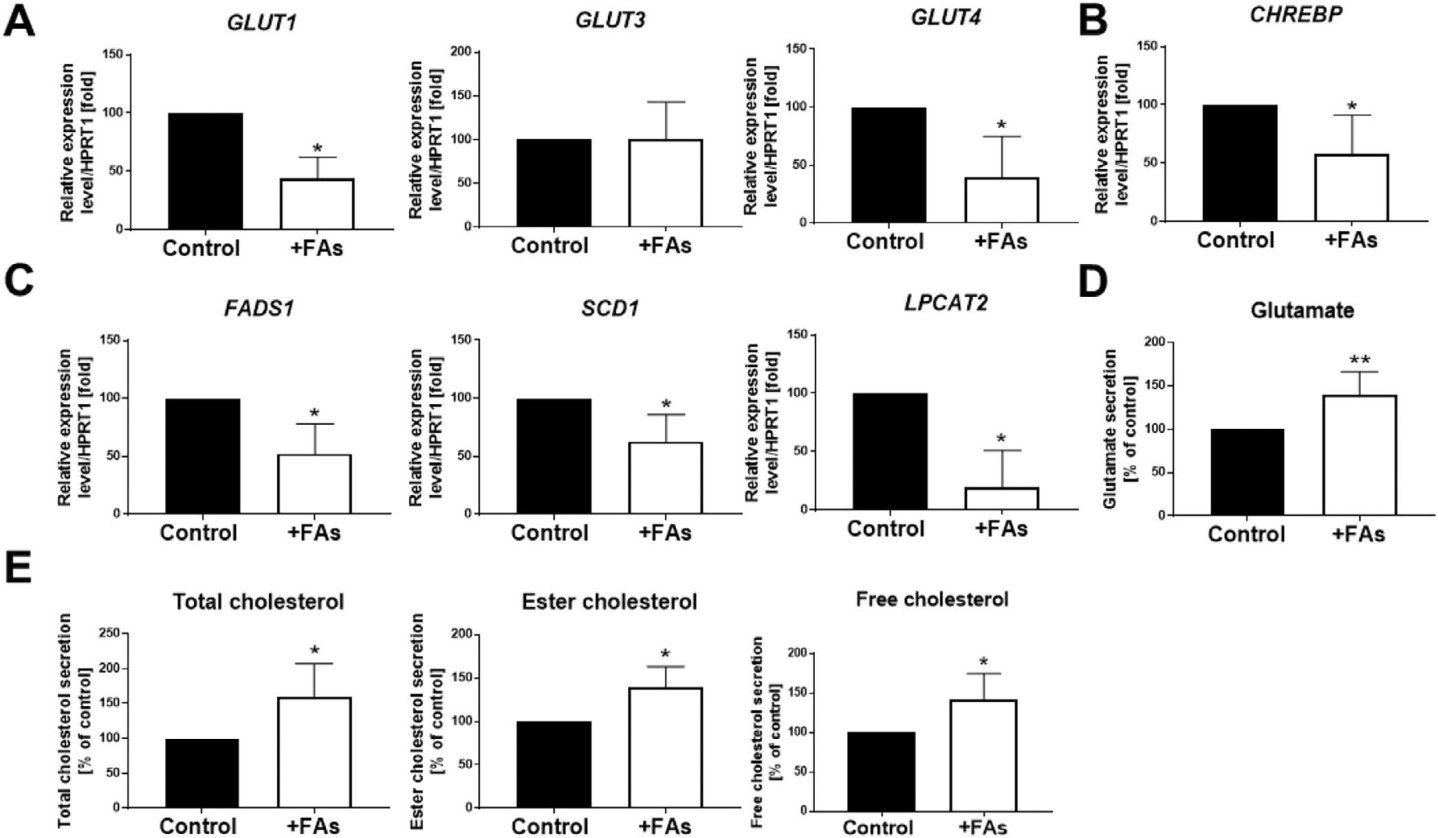
The impact of FAs on metabolism in adipocytes. mRNA expression levels of (A) glucose transporters: *GLUT1, GLUT3* and *GLUT4*, (B) *carbohydrate response element‐binding protein (CHREBP*), (C) *fatty acid desaturase 1 (FADS1), stearoyl‐CoA desaturase 1 (SCD1), lysophosphatidylcholine acyltransferase 2 (LPCAT2)* were determined by qRT‐PCR and normalised against the expression of *HPRT1*. The levels of secreted (D) glutamate, (E) total, ester and free cholesterol were measured using a chemiluminescent reaction in a cell‐conditioned medium. The results from at least three biological repetitions were calculated as the percentage of control and are presented as the mean ± SD. The significance level was set at *p* ≤ 0.05 (*), *p* ≤ 0.01 (**).

Fatty acids present in the cellular milieu may affect cellular glucose metabolism. Hence, we evaluated the expression level of glucose transporters. qRT‐PCR analysis showed that FAs lead to decreased mRNA expression of *GLUT1* and *GLUT4* but not *GLUT3* transporters in adipocytes (Figure 5A). Moreover, the mRNA expression level of the glucose‐sensing transcription factor—*carbohydrate response element‐binding protein* (*CHREBP*) was also downregulated by FAs (Figure 5B). After entering the cell, FAs can undergo various transformations, including esterification, elongation or desaturation [24] and these processes can be regulated by CHREBP [26]. A significantly reduced mRNA expression of *fatty acid desaturase 1* (*FADS1*) and *stearoyl‐CoA desaturase 1* (*SCD1*) was noted. Fattened adipocytes were also characterised by a lower level of *lysophosphatidylcholine acyltransferase 2* (*LPCAT2*) mRNA than in the control (Figure 5C), which catalyses the formation of phosphatidylcholine [27]. We also verified the secretion of metabolites in examined cells. Adipocytes incubated with FAs secreted higher amounts of glutamate (Figure 5D), as well as cholesterol (total, ester and free forms) (Figure 5E) in comparison to control cells. Overall, these findings suggest that FAs‐dependent hypertrophy of adipocytes induced the altered profile of secreted metabolites (glutamate and cholesterol) as well as affects glucose uptake, which in turn reflects in decreased mRNA levels of enzymes regulating lipid metabolism.

**FIGURE 6:**
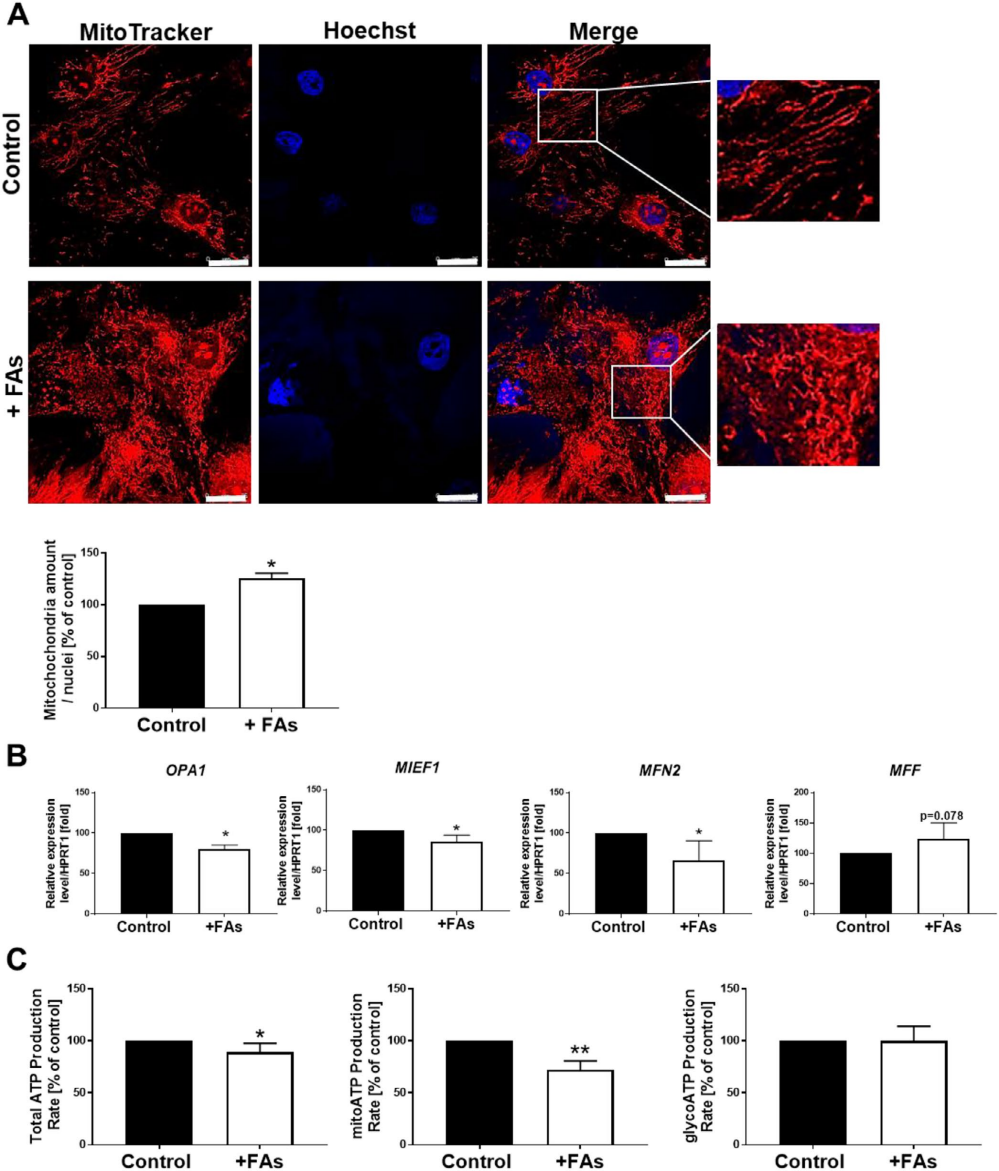
Mitochondrial changes in adipocytes promoted by FAs. (A) Representative images of cells stained for mitochondria (red) and cell nuclei (blue) with quantification of fluorescence intensity corresponding to mitochondria amount. Enlargement of the boxed, mitochondria‐rich areas are shown as insets. Scale bar: 25 μm. (B) qRT‐PCR assessment of *OPA1 (optic atrophy 1), MIEF1 (mitochondrial elongation factor 1), MFN2 (mitofusin 2)* and *MFF (mitochondrial fission factor)* mRNA expression level normalised against the mRNA expression of *HPRT1*. (C) Total‐, mito‐ and glycoATP production rate calculated based on the Seahorse oxygen consumption rate (OCR) and the extracellular acidification rate (ECAR) measurements. All results are presented as the mean ± SD from at least three independent experiments and were calculated as the percentage of control. *p* ≤ 0.05 (*), p ≤ 0.01 (**).

Next, we evaluated the impact of FAs on mitochondria distribution in adipocytes, since these organelles play a key role in maintaining lipid homeostasis and metabolism. The confocal microscopy images showed that in control adipocytes mitochondria formed thin, long fibres, whereas in fattened cells they were arranged in short, thick clusters dispersed in the entire cytoplasm (Figure 6A). Interestingly, quantitative analysis demonstrated that except for the alterations in the distribution of mitochondria, FAs induced an increase in their amount in adipocytes (Figure 6A). Considering the importance of mitochondrial dynamics in cellular functions, the expression of genes encoding proteins involved in the fusion and fission of that organelle was tested. qRT‐PCR results indicated that obtained hypertrophic adipocytes had downregulated mRNA expression of *OPA1* (*optic atrophy 1*), *MIEF1* (*mitochondrial elongation factor 1*) and *MFN2* (*mitofusin 2*) genes involved in mitochondria fusion, as well as slightly elevated mRNA expression of *MFF* (*mitochondrial fission factor*), which takes part in the process of their fission (Figure 6B). Since mitochondria are directly involved in the cellular metabolism, the real‐time ATP production was measured in the living cells. Fattened adipocytes exhibited lower total as well as mitoATP production rates (Figure 6C). We did not detect any alterations in the glycolysis‐based ATP production level (Figure 6C). Therefore, FA‐induced mitochondrial fragmentation in adipocytes could lead to reduced oxidative capacity and finally be connected with decreased ATP levels dependent on these organelles.

## 4 Discussion

Adipose tissue plays a significant role in whole‐body energy homeostasis. Under conditions of constant energy excess, adipocytes become hypertrophic and AT undergoes hyperplasia to increase their lipid storage capacity, as well as maintain circulating blood glucose and FA levels below the toxic value. However, adipocytes have a saturation point, where they lose the facility to store more lipids. At this stage, when adipocytes are lipid overloaded, they change their architecture and express stress signals [28]. The study of the disease mechanisms in obese adipocytes is a huge experimental challenge. The utilisation of adipocytes directly derived from patient biopsies is highly problematic because of the multi‐cellular complexity of AT and the characteristics of mature adipocytes; thus, in our study, we decided to rely on preadipocytes isolated from this tissue. Several experimental models have been generated to evaluate the mechanisms of obesity. A widely used model is the one based on the incubation of cells with FAs, which can be used in various compositions and concentrations. Palmitate was demonstrated to induce hypertrophy and endoplasmic reticulum stress in immortalised 3T3‐L1 adipocytes [29]. However, palmitate treatment induced not only more LD numbers and hypertrophy but also DNA damage and a senescent phenotype of human adipocytes [11]. Oleic acids enhanced the changes in cell morphology and lipid accumulation in 3T3‐L1 preadipocytes during the differentiation process by increasing the expression of C/ebpα (CCAAT/enhancer‐binding protein α) and Pparγ (peroxisome proliferator‐activated receptor γ) promoters [30]. However, most of the studies exploring the mechanisms of obesity‐related enlargement of adipocytes focus on the impact of only one type of FAs on mouse fibroblast‐derived adipocytes. We believe that this is not the appropriate approach because, firstly, it does not even partially reflect the diversity of FAs present in the patient's plasma, and secondly, the use of each of the mentioned FAs has a different effect on adipocytes. Pieters et al. [31] demonstrated that palmitic or oleic acids lead to the occurrence of such obesity‐related characteristics as a LD hypertrophy and insulin resistance, whereas only palmitic acid was able to increase adipocyte basal lipolysis and induce a shift in adipocyte secretome, making it capable of altering macrophage gene expression. Similar results were obtained by Mantilla‐Mora et al. [8]. As a third FA, linoleic acid was chosen, as it was demonstrated that it induces obesity and insulin resistance in mice to a greater extent than saturated acids [32]. We think that a very important voice in this discussion is the one described by Watt et al., who indicated that plasma contains a variety of long‐chain FAs, such that about 35% are saturated and 65% are unsaturated. They also point out that countless examples show how different FAs impart specific and unique effects, or even opposing actions, on cellular function; but despite these differing effects, palmitate (C16:0) is regularly used to represent ‘FAs’ in cell‐based experiments. Although palmitate can be useful to induce and study stress effects in cultured cells, these effects in isolation are not physiologically relevant to dietary manipulations, obesity or the consequences of physiological concentrations of FAs [27]. This approach is problematic because palmitate often induces cytotoxic responses that are at variance with most other FAs. It is observed in many cases that the addition of an equal concentration of oleate prevents the adverse effects of palmitate. Thus, it is incorrect to incubate cells with palmitate in the absence of other unsaturated FAs and infer that the outcome represents a physiological effect of ‘FAs’ or of ‘saturated FAs’. To avoid mistaken interpretations, Watt et al. recommend that FAs should be used at physiological concentrations (e.g., 50–750 μM; in our case it is 170–330 μM). They also suggest that investigators should perform experiments with a mixture of saturated and unsaturated FAs delivered in a molar ratio and in concentrations that represent those found in blood (e.g., 1:1 or 1:2 palmitate/oleate; 1:2:1 palmitate/oleate/linoleate) [27]. It is worth mentioning that in humans, the most abundant FAs esterified to triacylglycerols (TAG) are oleate (C18:1n9), palmitate (C16:0) and linoleate (C18:2n6). These three FAs constitute approximately 85% of all TAG [33]. This is the reason why, in our work, we decided to generate artificially hypertrophied cells by incubating human adipocytes with a mixture of linoleate, palmitate and oleate, which, in our opinion, better reflects the in vivo conditions.

The hallmark of adipocytes' hypertrophy is an increase in LDs content [5]. This feature was also detected in our model, where the FAs induced the accumulation of LDs. Perilipins (PLINs) are LD‐associated proteins controlling their size and hydrolysis. Under basal conditions, PLINs restrict the access of cytosolic lipases to LDs and thus promote triacylglycerol storage [18]. Although PLIN1 is the predominant form produced in adipocytes, PLIN2 is expressed ubiquitously [34]. Interestingly, PLIN2 is present on the surface of lipid‐poor small LDs, and it is replaced by PLIN1 in lipid‐rich large particles. Hence, PLIN1 may be involved in the formation of large unilocular LDs (often larger than 10 μm) [35]. This may explain our observation demonstrating enrichment of large (> 100 μm^2^) LDs population in fattened adipocytes associated with increased expression of PLIN1. Moreover, we also observed a higher amount of small LDs, which may be connected to the expression of PLIN2 at a non‐reduced level.

Several possible ways by which cells can take up lipids were described. Translocation across the plasma membrane by specific transporters, apart from passive diffusion, is one of them [19]. CD36 is a high‐affinity receptor for long‐chain FAs that contributes to lipid accumulation and metabolic dysfunction under conditions of excessive fat supply [36]. What is more, CD36 appears to be the predominant membrane protein facilitating FA transport—in AT of mice with a targeted deletion of this transporter, reduced FA uptake rates (by 60%–70%) were shown [37]. We detected that the excess of FAs in the cellular milieu led to the upregulation of CD36 as well as FABP4 and FABP5. FABP expression was also elevated in the AT of obese patients [38]. FAs that are taken up by cells are then bound to FA‐binding proteins (FABPs), which help in their intracellular transport and affect lipid and glucose metabolism by increasing FAs levels [19].

The circulating levels of adiponectin were inversely correlated with obesity [39]. However, the in vitro data are inconsistent. Karki et al. [40] have found that palmitate inhibits transcription of the adiponectin gene and reduces the release of this protein from 3T3‐L1 adipocytes. However, another group noted that hypertrophic adipocytes (also derived from 3T3‐L1 fibroblasts) achieved by exposition to oleate and palmitate presented upregulated expression of this adipokine [41]. This is in line with our results, where adipocytes incubated with the mixture of FAs expressed and secreted a higher level of adiponectin. In fully differentiated adipocytes, adiponectin‐overexpressing cells accumulated more and larger LDs compared with control cells [42]. Adiponectin expression was demonstrated to be regulated by CD36 [20]. Conversely, this adipokine was also shown to enhance the surface expression of CD36 in human taste cells, thus selectively improving their response to FAs [43]. There may exist a feedback loop between these two molecules; however, it needs further evaluation.

Adipocyte size changes as a result of a pronounced accumulation of LDs, which occupy most of the adipocyte volume. This is associated with a remodelling of intracellular architecture and compression of other organelles. Bearing this in mind, we analysed vimentin and actin distribution in FA‐treated adipocytes. Vimentin takes part in the formation of LDs in human adipocytes. This protein was described to form a scaffold around LDs, which is achieved by interactions of vimentin with perilipins [44]. Depletion of vimentin resulted in smaller adipocytes and LDs [22], which indicates the direct involvement of this intermediate filament protein in the lipid storage process. In our study, supplementation with FAs led to the overproduction of vimentin as well as its rearrangement in adipocytes. This may be connected with the need to support the structure of extremely big LDs as well as, at least partially, to overcome the mechanical stress induced by the intense increase of lipid content. Atomic force microscopy analysis identified that adipocytes with accumulated large LDs were more compliant and fluid‐like [45], which may be reflected also in the cytoskeletal remodelling of the actin network. One could speculate that the observed shift in F:G actin ratio in LDs‐overloaded adipocytes is a mechanism to stabilise a more fluid‐like cell body. This hypothesis is supported by the reports of other groups. The expansion of primary adipocytes in mice fed a high‐fat diet for 2 weeks was associated with a drastic increase in the F‐actin pool and changed expression of actin‐regulating proteins, favouring actin polymerisation [23]. In skeletal muscle cells, palmitate abrogated actin remodelling and increased cell stiffness [46]. Moreover, palmitic acid or high‐fat diet promoted interaction between CD36 and Src kinase leading to its activation, which finally resulted in actin network remodelling in lung adenocarcinoma cells [47]. Src family kinases play key roles in the regulation of signal transduction in a variety of cellular processes, including the differentiation of human preadipocytes [48] as well as the dynamics and organisation of vimentin filaments [49]. Therefore, the detected alteration in Src phosphorylation (inducing its activation) may be one of the factors affecting the actin polymerisation state but also the formation of vimentin filaments in fattened adipocytes [25].

Apart from the accumulation of LDs, which enlarge dramatically during FA excess, as well as connected with it the reorganisation of cell intracellular architecture, adipocyte hypertrophy is also associated with a wide spectrum of metabolic dysregulation. Over‐nutrition of adipocytes by high‐glucose or a palmitic acid‐supplemented milieu led to reduced expression of the glucose receptor—GLUT4 [50]. However, Bolsoni‐Lopes et al. [51] demonstrated that palmitoleic acid increases the glucose uptake and the GLUT4 content in association with AMPK activation. Oleic and linoleic FAs were indicated to downregulate GLUT4 expression in skeletal muscle cells [52]. In light of the incoherent data, we also evaluated the mRNA expression level of glucose transporters. Our results clearly showed the downregulation of *GLUT1* and *GLUT4* transporters as an effect of the overloading of adipocytes with FAs. Both of them are implicated in the entering of glucose into cells. A consequence of the likely decrease in intracellular glucose concentration may be a diminished expression level of CHREBP. This transcription factor is a nutrient‐sensing (especially glucose) regulator, indispensable for the expression of a diverse range of metabolic genes, including the lipogenic enzymes necessary for the metabolic conversion of carbohydrates into lipids [53]. Dentin et al. [54] claimed that polyunsaturated FAs inhibit CHREBP by decreasing its nuclear localization. It is possible that in our experimental model the reduced level of *CHREBP* may be connected with the decreased levels of enzymes regulating lipid metabolism—*FADS1*, *SCD1* and *LPCAT2*. The main pathway by which FAs are catabolised is β‐oxidation, which occurs in mitochondria and peroxisomes. SCD‐ and FADS‐dependent FA desaturation pathways coexist in cells [24], and the mRNA expression of both of these enzymes was reduced by FA exposure. On the other hand, hypertrophy of adipocytes induced by over‐nutrition may be reflected in the altered profile of secreted metabolites. Obesity was previously reported to elevate glutamate concentrations in white AT [55]. We detected increased glutamate and cholesterol levels in media collected from fattened adipocytes. As adipocyte hypertrophy progresses during obesity development, cellular cholesterol content increases [56]. A specific feature of adipocyte cholesterol, in contrast to other cell types, is the relative lack of its esterified form (> 95% of adipose cholesterol consists of free cholesterol), which accumulates in LDs [57]. Thus, it is probable that FA‐overloaded adipocytes try to maintain cholesterol homeostasis by cholesterol efflux, which, at least partially, would explain the higher secretion of free cholesterol. However, the reason for increased cholesterol ester efflux by adipocytes incubated with FAs is not clear and needs additional investigation.

The main function of mitochondria is the maintenance of energy homeostasis. High‐fat diet feeding of mice caused mitochondrial fragmentation in adipocytes, leading to reduced oxidative capacity [58]. A similar phenomenon, with a concomitant rise in mitochondria amount, was observed by us in human adipocytes incubated with FAs. Other groups also reported that overloaded with palmitic acid hypertrophic adipocytes [41] exhibited altered mitochondrial status. Palmitate treatment of hepatocytes induced downregulation of MFN2 in both transcript and protein levels [59]. Moreover, high‐glucose/high‐fat‐treated cardiomyocyte mitochondria became fragmented and the expression of MFN2 was significantly reduced [60], whereas this protein was shown to suppress the accumulation of LDs and the progression of clear cell renal cell carcinoma [61]. Our results indicate that in hypertrophic adipocytes, FAs treatment induces the downregulation of mRNA expression of *OPA1*, *MIEF1* and *MFN2* genes responsible for mitochondrial fusion. Interestingly, OPA1 was recently also shown to be located on LDs in adipocytes, where it functions as an A‐kinase anchoring protein that mediates adrenergic control of lipolysis by facilitating phosphorylation of PLIN1 [62]. The dynamic cycling of mitochondrial fusion and fission is essential for maintaining various cellular functions. Obesity as well as excess energy intake shift the balance of mitochondrial dynamics, further contributing to mitochondrial dysfunction and metabolic deterioration. A similar situation was observed in our model, where despite the increased amount of mitochondria, their probable dysfunctionality led to the decreased ATP production rate in fattened adipocytes. Interestingly, Wessels et al. [63] noted that obesity is the primary driver of impaired adipocyte mitochondrial respiration. The association between mitochondrial morphology and LD accumulation in response to high exogenous FAs was observed [64]. Potes et al. [65] reported that the fusion mechanism was also affected by being overweight, with a concomitant decrease in the protein levels of DRP1 and FIS1. Src, whose phosphorylation level was altered in FA‐treated adipocytes, also plays a role in the regulation of mitochondrial dynamics. Lurette et al. [66] observed that the deletion of Src kinase decreased the percentage of mouse embryonic cells with tubular mitochondria and increased the number of cells with elongated mitochondria, which was accompanied by their decreased number and increased area of individual organelles. Therefore, obesity appears to affect the regulation of mitochondrial dynamics, disturbing the usual mechanism of division and fusion, which affects its balance.

## 5 Conclusions

Here, we describe the effect of treating human primary adipocytes with a mixture of FAs. The use of such experimental conditions allows us to closely imitate the model of human obesity. Fattened adipocytes formed significantly more LDs and had upregulated expression of proteins promoting triacylglycerol storage. They also produce at a higher level proteins involved in the transport of FAs into the cell and within it. Additionally, lipid‐overloaded adipocytes secreted more adipokines, cholesterol and glutamate, which can affect surrounding cells as signalling molecules or as a potential source of energy. The response of adipocytes to FAs treatment was also visible in the elevated level of vinculin and actin polymerisation, which can help to stabilise both large LDs and a more fluid‐like cell body. At the same time, these cells exhibited a raised number of mitochondria and decreased expression of genes related to their fusion compared to control adipocytes, which may indicate their dysfunction. In conclusion, this work contributes to the understanding of molecular mechanisms involved in human adipocyte fattening and may thus help in developing new methods of treating obesity‐related ailments.

## Author Contributions

**Katarzyna Pietraszek‐Gremplewicz:** conceptualization (lead), formal analysis (lead), investigation (lead), writing – original draft (lead), writing – review and editing (lead). **Joanna Olszańska:** investigation (supporting), writing – review and editing (supporting). **Mikołaj Domagalski:** formal analysis (supporting), visualization (equal), writing – review and editing (supporting). **Aleksandra Simiczyjew:** writing – review and editing (supporting). **Magdalena Kot:** writing – review and editing (supporting). **Aneta Skoniecka:** resources (supporting), writing – review and editing (supporting). **Agata Tymińska:** resources (supporting), writing – review and editing (supporting). **Michał Pikuła:** resources (supporting), writing – review and editing (supporting). **Dorota Nowak:** funding acquisition (lead), supervision (lead), writing – review and editing (supporting).

## Ethics Statement

In this study, AD‐MSCs isolated from human subcutaneous AT were used. The tissue was obtained from patients at a plastic surgery clinic (Gdansk, Poland). All procedures were conducted following informed consent from patients and with the approval of the Bioethics Committee for Scientific Research of the Medical University of Gdansk (NKBBN/858/2022‐2023). The tissue samples were considered medical waste following aesthetic body contouring procedures.

## Conflicts of Interest

The authors declare no conflicts of interest.

## Data Availability

Data will be made available on request.
